# Balloon-assisted, ultrasound-guided percutaneous real-time thrombin injection, for the arteriovenous fistula pseudoaneurysm treatment

**DOI:** 10.1016/j.radcr.2022.10.074

**Published:** 2022-11-24

**Authors:** Giuseppe Giordano, Oriana Roberta Sollami, Diego Meo, Sebastiano Piana, Viviana Lentini, Vincenzo Magnano San Lio

**Affiliations:** Unit of Diagnostic and Interventional Radiology ARNAS “Garibaldi–‐Nesima”, Via Palermo 636, 95122, Catania, CT, Italy

**Keywords:** Arteriovenous fistula, Pseudoaneurysm, Thrombin

## Abstract

A pseudoaneurysm or false aneurysm is the consequence of a persistent blood leak caused generally by iatrogenic rupture of a vessel wall. The blood leak creates a new cavity delimited by surrounding tissues and allows blood flow to remain in continuity between this cavity and the vessel. In hemodialysis fistula, pseudoaneurysm generally results from repeated puncturing of the vein at the same site, leading to a bulging anatomical defect in the vein. Over the past few years, interventional radiological treatment has evolved and taken the place of surgery, with different kinds of percutaneous and endovascular treatment methods in pseudoaneurysm management. We reported a case report of successful treatment of arteriovenous fistula pseudoaneurysm with no-measurable neck. We performed ultrasound-guided percutaneous direct thrombin injection while an inflated balloon transiently obstructed flow out of the pseudoaneurysm, in order prevent non-target embolization.

## Case report

A 79-year-old man with end-stage renal disease undergoing hemodialysis with a radio-cephalic arteriovenous fistula (AVF). Nine months after fistula creation, the patient arrived to our attention because of the presence of a post-anastomotic hemodynamic venous stenosis.

**Fig. 1:**
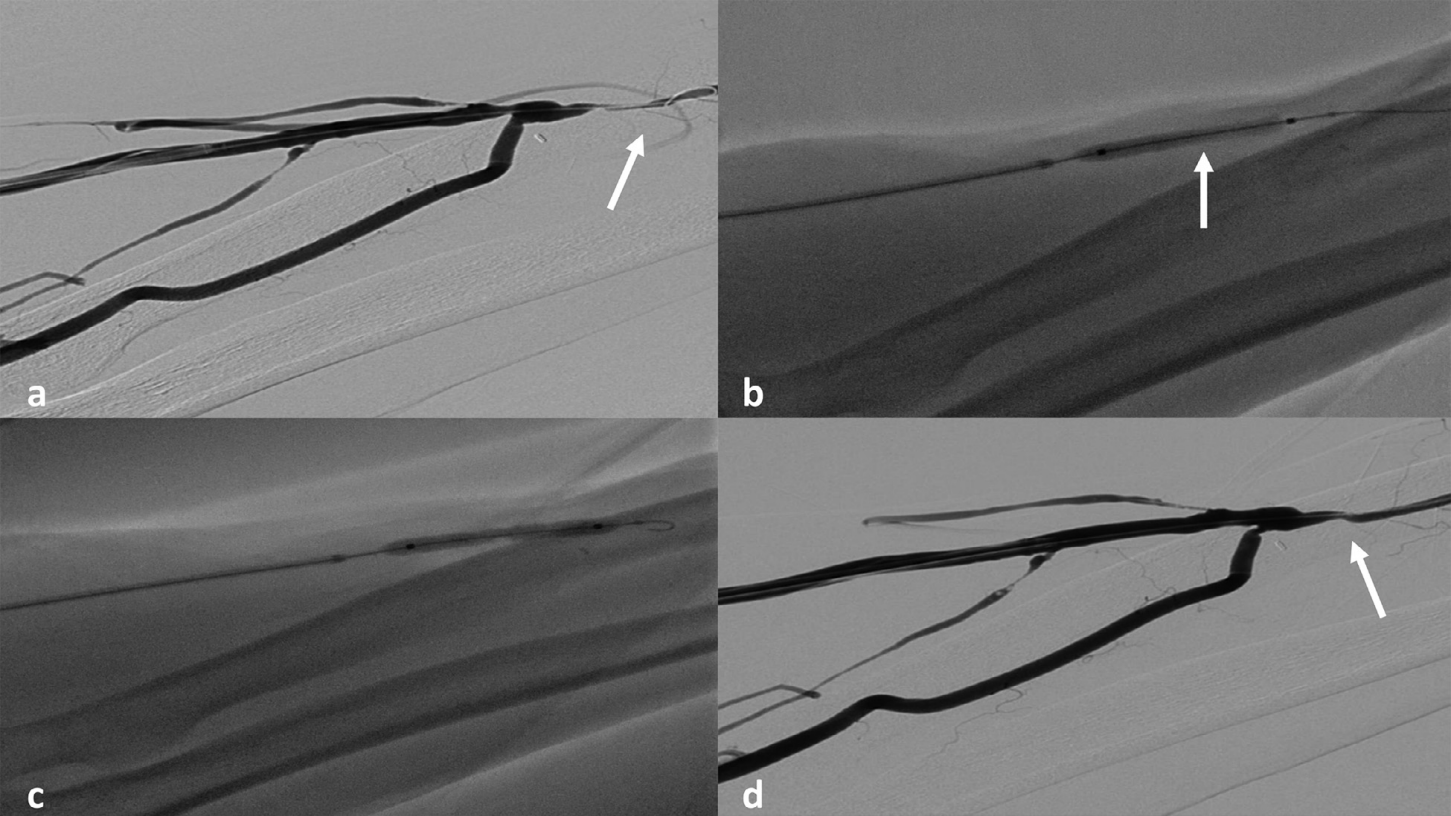
The diagnostic angiography confirmed a focal post-anastomotic stenosis (a; arrow) that was treated with a balloon (b and c) with good angiographic result (d).

Under local anesthesia, an ultrasound-guided puncture of AVF was performed and a 5F sheath was positioned in the draining vein. The diagnostic angiography confirmed a focal post-anastomotic stenosis that was treated with a 5 mm × 40 mm balloon with good angiographic result (Fig. 1).

A month after, as a result of this procedure, the patient presented just after her hemodialysis session, a pulsatile and painful mass localized in cannulation site, so a new diagnostic angiography was performed and it was confirmed the presence of wide-necked pseudoaneurysm in the draining vein, at the level of previous puncturing site.

**Fig. 2:**
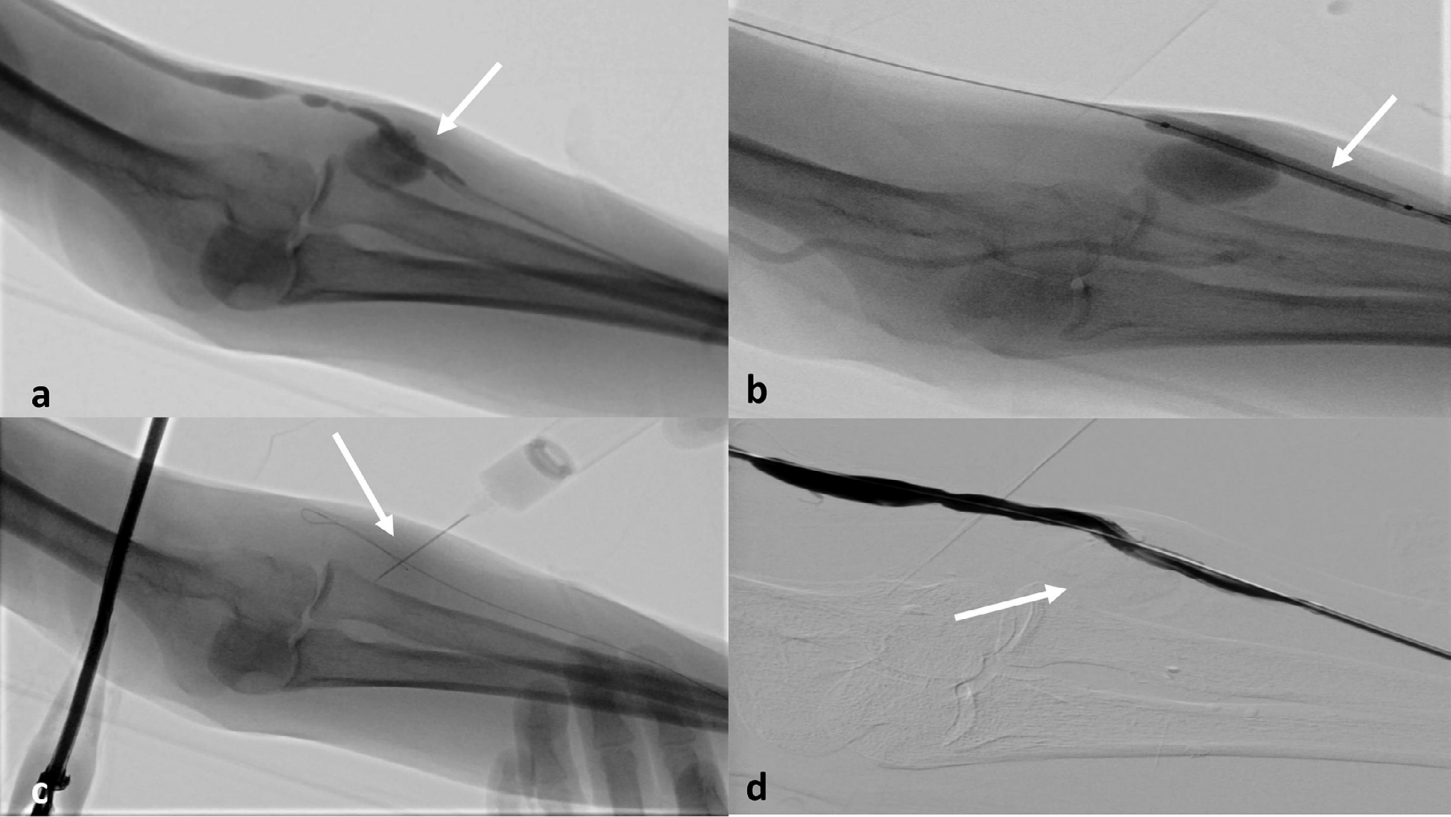
The diagnostic angiography confirmed the presence of pseudoaneurysm in the draining vein (a). A balloon was positioned and inflated across the pseudoaneurysm basis (b) and then, under ultrasound guidance, the pseudoaneurysm was punctured with a 21-gauge needle and 2 mL of thrombin was real-time injected, until to complete filling the pseudoaneurysm (c). At the final check, the pseudoaneurysm was completely excluded from the circulation (d).

A 6 × 60 mm balloon was advanced over the wire and positioned and inflated across the pseudoaneurysm basis in order to exclude the cavity from the circulation then, under ultrasound guidance, the pseudoaneurysm was punctured with a 21-gauge needle and 2 mL of thrombin was real-time injected under ultrasound guidance, until to complete filling the pseudoaneurysm (Fig. 2). The balloon was then deflated.

Flebography demonstrated, also, a juxta-anastomotic stenosis, that was treated with a 6 mm × 60 mm balloon with good angiographic result.

At the final check, there was no residual stenosis, while the pseudoaneurysm was completely excluded from the circulation.

## Discussion

Depending on size and location, it is reported in literature that some pseudoaneurysms may undergo spontaneous thrombosis, as the majority of small post-catheterization pseudoaneurysms [1,2]. Based on these reports, conservative management may be recommended in asymptomatic iatrogenic femoral artery pseudoaneurysms. However, some departments prefer treating iatrogenic femoral artery pseudoaneurysms in case any complication occurs [3].

Many kinds of endovascular or percutaneous methods are described for pseudoaneurysm treatment, such as ultrasound-guided compression, covered stent, detachable balloon, coil embolization, or percutaneous thrombin injection [4].

However, while for wide-necked pseudoaneurysm treatment, it also reported thrombin injection under ultrasound guidance with the use of covered stent deployed across the neck of the pseudoaneurysm [5], very few cases are reported in literature of pseudoaneurysm treatment with balloon-assisted, ultrasound-guided percutaneous real-time thrombin injection [6].

In this case, ultrasound-guided local thrombin injection has the advantage of being a low cost, to guarantee the effectiveness of treatment, not needing sedation, less traumatic, technically simple procedure in experienced hands, paying highly attention to avoid non-target embolization [7]. Moreover, thrombin reduces significantly the presence of artifacts at the CT scan and gives us the exact extension of thrombosis into the aneurysm sac, while the ultrasound-guided offers the advantage of monitoring the progression of the thrombotic process induced by thrombin injection in real time [8], without X-rays use.

Finally, the absence of a covered stent across puncturing site guaranteed AVF long-term patency: after 1 month from the treatment, the patient continued to dialyze successfully with no further complications.

## Patient consent

Informed consent was obtained from all individual participants included in the study. Additional informed consent was obtained from all individual participants for whom identifying information is included in this article.

## Ethical approval

Our institution does not require ethical approval for reporting individual cases or case series. All procedures performed in studies involving human participants were in accordance with the ethical standards of the institutional and/or national research committee and with the 1964 Helsinki declaration and its later amendments or comparable ethical standards.
